# CoeViz 2: Protein Graphs Derived From Amino Acid Covariance

**DOI:** 10.3389/fbinf.2021.653681

**Published:** 2021-06-24

**Authors:** Daniel Corcoran, Nicholas Maltbie, Shivchander Sudalairaj, Frazier N. Baker, Joseph Hirschfeld, Aleksey Porollo

**Affiliations:** ^1^ Department of Electrical Engineering and Computing Systems, University of Cincinnati, Cincinnati, OH, United States; ^2^ Advanced Concepts Laboratory, Georgia Tech Research Institute, Fairborn, OH, United States; ^3^ Center for Autoimmune Genomics and Etiology, Cincinnati Children’s Hospital Medical Center, Cincinnati, OH, United States; ^4^ Division of Biomedical Informatics, Cincinnati Children’s Hospital Medical Center, Cincinnati, OH, United States; ^5^ Department of Pediatrics, University of Cincinnati College of Medicine, Cincinnati, OH, United States

**Keywords:** amino acid covariance, coevolving functional sites, protein ligand binding sites, protein molecular graph, CoeViz

## Abstract

Proteins by and large carry out their molecular functions in a folded state when residues, distant in sequence, assemble together in 3D space to bind a ligand, catalyze a reaction, form a channel, or exert another concerted macromolecular interaction. It has been long recognized that covariance of amino acids between distant positions within a protein sequence allows for the inference of long range contacts to facilitate 3D structure modeling. In this work, we investigated whether covariance analysis may reveal residues involved in the same molecular function. Building upon our previous work, CoeViz, we have conducted a large scale covariance analysis among 7,595 non-redundant proteins with resolved 3D structures to assess 1) whether the residues with the same function coevolve, 2) which covariance metric captures such couplings better, and 3) how different molecular functions compare in this context. We found that the chi-squared metric is the most informative for the identification of coevolving functional sites, followed by the Pearson correlation-based, whereas mutual information is the least informative. Of the seven categories of the most common natural ligands, including coenzyme A, dinucleotide, DNA/RNA, heme, metal, nucleoside, and sugar, the trace metal binding residues display the most prominent coupling, followed by the sugar binding sites. We also developed a web-based tool, CoeViz 2, that enables the interactive visualization of covarying residues as cliques from a larger protein graph. CoeViz 2 is publicly available at https://research.cchmc.org/CoevLab/.

## Introduction

Amino acid covariance analysis is a widely used approach to identify protein residues coevolving through different species. Such coevolving positions are frequently used to impose constraints in protein 3D structure modeling, assuming the driving force of coevolution for such positions is due to their direct contacts and steric constraints in the folded state (Marks et al., 2012; de Juan et al., 2013). On the other hand, different evolutionary forces, such as involvement in the same molecular function (MF), are largely understudied.

Covariance analysis utilizes multiple sequence alignment to compute couplings between pairs of positions in a protein sequence. These couplings essentially represent frequencies of observing a certain type of amino acid at a given position together with the other amino acid at another position, computed across all aligned sequences. Some methods also consider gap in the aligned sequence as an additional amino acid type. These couplings are then used to compute various covariance scores, e.g., mutual information.

Many efforts in covariance analysis, collectively called the direct-coupling analysis (DCA), are dedicated to distinguishing the direct from indirect couplings of positions. Representative methods include mpDCA (Weigt et al., 2009), mfDCA (Morcos et al., 2011) and plmDCA (Ekeberg et al., 2013) that employ message passing, mean-field approximation and pseudo-likelihood maximization algorithms, respectively, to eliminate indirect couplings. Another DCA method, PSICOV uses the graphical LASSO algorithm (Friedman et al., 2008) to obtain the estimated sparse matrix inverse of the covariance matrix, indicative of the significance of empirical couplings (Jones et al., 2012). Thus adjusted couplings are then used to compute Direct Information (DI) or PSICOV (PC) scores, respectively.

With the variety of currently available amino acid covariance methods, the field demonstrates certain trends. 1) Due to the complexity of statistical models and large matrices of couplings (e.g., 21L x 21L, where L is the protein sequence length) employed, only single domain protein sequences are considered, limiting studies to relatively short sequences. 2) In an effort to accelerate calculations, average product correction [APC, (Dunn et al., 2008)] is used to adjust the resulting covariance matrices for entropic bias (Jones et al., 2012; Zerihun et al., 2020) as opposed to time consuming weighting of each alignment to account for phylogenetic bias while computing position-specific probabilities (Weigt et al., 2009). 3) Most applications are geared toward the identification of inter-residue contacts in the context of 3D structure modeling, while the coevolution of functional residues that are not necessarily in direct contact is underexplored, especially on a large scale.

In this work, we explored whether three different covariance metrics can reveal the groupings of residues by MF and distinguish them from other residues. Building upon our previous implementation (Baker and Porollo, 2016), the covariance matrices were compared with those processed using graphical LASSO to obtain estimated covariance matrix and its inverse, and the state-of-the-art covariance estimation DCA methods. To visualize protein sequence as a molecular graph based on covariance data and to explore residues forming cliques, we developed a web-based tool CoeViz 2. In contrast to other existing tools, CoeViz 2 can deal with full length sequences, not just single domains.

## Materials and Methods

### Benchmark Set

The protein set with annotated functional residues was compiled from the BioLiP database (Yang et al., 2013) using the following criteria:

• A protein sequence has a reference to UniProt to avoid synthetic or chimeric sequences.
• A protein structure binds a natural ligand.
• Sequence length is at least 30 amino acids long.
• Sequence alignment against the UniProt UniRef90 database yields at least 100 homology hits.
• Sequence identity between proteins within the set is less than 25%.

Residues binding natural and most frequently occurring ligands in the dataset were considered functional sites. The actual list of proteins used herein along with sequences and annotations can be downloaded from the home page of CoeViz 2.

Amino acid composition among ligand binding sites was assessed using the observed frequencies normalized to the background distribution of amino acids as follows:

$$P\left(a|l\right)=\log\;\frac{f_{a,l}}{F_{a}}$$ (1)

where *P(a|l)* is propensity of the amino acid type *a* to bind the ligand *l*; *f*_*a,l*_ is its corresponding fraction among the all types of residues found to bind this ligand across all considered proteins; and *F*_*a*_ is the overall frequency of a given amino acid in the assessed protein set.

### Covariance Metrics

Covariance matrices have been generated as described in (Baker and Porollo, 2016). Specifically, mutual information (MI, Eq. 2), χ^2^– (Eq. 3), and Pearson correlation (*r*)-based (Eq. 4) metrics were computed based on multiple sequence alignments (MSA) adjusted for phylogenetic bias (Eqs 5–7).

$$\mathrm{MI}\left(i,j\right)=\underset{x}{\sum }\underset{y}{\sum }p_{\mathrm{ij}}\left(x,y\right)\log\frac{p_{\mathrm{ij}}\left(x,y\right)}{p_{i}\left(x\right)p_{j}\left(y\right)}$$ (2)

$$\chi^{2}\left(i,j\right)=\underset{x}{\sum }\underset{y}{\sum }\frac{{\left(p_{\mathrm{ij}}\left(x,y\right)-p_{i}\left(x\right)p_{j}\left(y\right)\right)}^{2}}{p_{i}\left(x\right)p_{j}\left(y\right)}$$ (3)

$$r\left(i,j\right)=\frac{1}{N_{\mathrm{eff}}}\underset{l}{\sum }\frac{w_{\mathrm{sl}}\left(s_{\mathrm{il}}-{\bar{s}}_{i}\right)\left(s_{\mathrm{jl}}-{\bar{s}}_{j}\right)}{\sigma_{i}\sigma_{j}}$$ (4)

$$p\left(s\right)=\frac{w_{\mathrm{sl}}}{N_{\mathrm{eff}}+1}$$ (5)

$$N_{\mathrm{eff}}=\underset{l}{\sum }w_{\mathrm{sl}}$$ (6)

$$w_{\mathrm{sl}}^{a}={\left|\left\{b\in \left\{1,\ldots ,N\right\}\text{|}\mathrm{seqid}\left(A^{a},A^{b}\right)>80\%\right\}\right|}^{-1}$$ (7)

where *x* and *y* are the amino acid types at positions *i* and *j*, respectively, binned in two categories: 1 if it matches the amino acid at this position in the query sequence, and 0–otherwise; *p(s)* is the observed frequency of state *s* = {x; y; x,y}; *N*_*eff*_ is the effective sum of weights of alignments where both positions are not gaps. *w*_*sl*_ is a weighted count of state *s* of the alignment *l*, which, for the phylogeny weighting scheme used in this work, is a weight for sequence *A* ^*a*^ in the MSA of *N* total sequences that equals to one over the number of sequences *A* ^*b*^ in the MSA that have at least 80% sequence identity to *A* ^*a*^. 80% was chosen as a midpoint of the range 70–90%, where there is no strong dependence observed on the precise threshold value (Morcos et al., 2011). *s*_*il*_ is a similarity score that quantifies the change of an amino acid at position *i* to the one in the aligned sequence *l*. ${\bar{s}}_{i}$ and *σ*_*i*_ are mean and standard deviation, respectively, of all similarity scores of amino acid substitutions for a given position represented across the all sequences aligned to the query. Similarity scores are taken from the position specific similarity matrix (PSSM) generated by PSI-BLAST. Thus generated covariance matrices are subsequently processed as follows. The χ^2^ scores are converted into cumulative probabilities (df = 1). The *r* scores are taken by their absolute values.

In this work, for comparison purposes, a 20-letter alphabet (i.e. *x* and *y* are the sets of 20 natural amino acids) has also been implemented for MI and χ^2^ metrics, as an alternative to the original CoeViz *p(s)* calculations based on the 2-letter alphabet. Furthermore, all matrices are subjected to the sparse inverse covariance estimation (Eq. 8) using the fast implementation of graphical LASSO (R library glassoFast) (Friedman et al., 2008; Sustik and Calderhead, 2012).

$$\hat{\Theta }=\mathrm{argmin}\left(\underset{\mathrm{ij}}{\sum }S_{\mathrm{ij}}\Theta_{\mathrm{ij}}-\mathrm{logdet}\left(\Theta \right)+\rho \underset{\mathrm{ij}}{\sum }\left|\Theta_{\mathrm{ij}}\right|\right)$$ (8)

where *S* is the empirical covariance matrix, *Θ*–its inverse, *ρ*–regularization parameter (the higher its value is, the more zero elements are in the estimated inverse matrix $\hat{\Theta }$ ). To automate the process, *ρ* is chosen to have the minimal value at which both estimated covariance matrix and its inverse do not have undefined elements. This value is found using the binary search in the range (0.01, 0.99).

### Distributions of Covarying Functional Sites

For the functional residue at position *i*, enrichment for residues (*r*) involved in the same MF (*f*) among the all covarying positions (*n*) with covariance scores *cov(i,j)* ≥ given cutoff (*c*) is quantified by log odds (*LO*):

$$LO_{i}^{f}=\log\frac{\frac{r_{\mathrm{cov}\left(i,j\right)\geq c}^{f}}{R^{f}-1}}{\frac{n_{\mathrm{cov}\left(i,j\right)\geq c}}{N-1}}$$ (9)

where *R* ^*f*^ is the total number of residues with the MF *f* in a given protein, *N* is the total number of residues in the protein; both adjusted for the residue of interest.

Alternative scores *LO(q)* are computed based on the graph cliques:

$$\mathrm{LO}{\left(q_{k}\right)}_{i}^{f}=\log\frac{\frac{r{\left(q_{k}\right)}_{c}^{f}}{R^{f}-1}}{\frac{n{\left(q_{k}\right)}_{c}}{N-1}}$$ (10)

where *r* are residues involved in the same MF (*f*) among the all residues (*n*) in the *k*-th clique (*q*) derived for the given cutoff (*c*); *R* ^*f*^ and *N* are the same as in Eq. 9. Note, in this formulation, some functional sites may have no covariance scores with the residue of interest (*i*-th) above a given cutoff.

To quantify the overall enrichment for the residues involved in the same function by a given metric, the LO probabilities *P(LO)* are computed as 1–cumulative probability of the empirical LO density distribution for a given LO cutoff. Naturally, the larger *P(LO)* at a higher LO cutoff the stronger grouping of functional residues a given metric yields.

### Web Interface

CoeViz 2 provides an interactive interface to visualize molecular graphs, where nodes are amino acid residues colored according to their conservation scores (Shannon entropy), and edges are pairwise covariance scores. The tool enables identification of graph cliques by adjusting the covariance cutoff. For easier navigation, distributions of nodes, edges, cliques and clique sizes for a precomputed range of covariance cutoffs are provided as plots at the navigation panel. The user can also export the currently viewed graph as well as the underlying covariance matrix and the MSA. The navigation panel also contains a cross-link to CoeViz for the previously implemented visual analysis of covariance data, including interactive heatmaps, hierarchical trees, circular diagrams, and multi-dimensional scaling (MDS) 3D plots (Baker and Porollo, 2018).

CoeViz 2 is publicly available at https://research.cchmc.org/CoevLab/. The web interface of CoeViz 2 is based on Python modules pyviz for the interactive visualization of graphs using HTML5 canvas, and networkx for the definition of graphs and identification of cliques.

## Results

### Summary of the Benchmark Set

The benchmark set encompasses 7,595 non-redundant proteins, ranging from 30 to 3,005 aa. All natural ligands most frequently encountered in these proteins according to Protein Data Bank (PDB) (Berman et al., 2000) fall into seven categories (Table 1). DNA/RNA and metal binding sites appear to be the most abundant.

**TABLE 1:** Functional sites used for benchmarking.

Category	PDB ligand coding	Total residues
DNA/RNA	NUC	27,381
Metal	CA, CU, FE, FE2, MG, MN, ZN	25,548
Dinucleotide	NDP, NAD, NAP	11,130
Nucleoside	ADP, AMP, ATP, GDP	9,667
Heme	HEC, HEM	9,467
Sugar	BGC, FUC, GAL, GLC, MAN, NAG	3,457
Coenzyme A	COA	1,994

Amino acid frequencies per ligand, normalized to the background amino acid composition in the benchmark set (Eq. 1), are summarized in Table 2. Cys (C) appears to have the highest propensity to bind metals, and to a lesser degree heme. Asp (D) is also frequently observed to bind metal ions, while having strong negative preference to coenzyme A and heme. Glu (E) is very rarely observed to bind coenzyme A, heme, and dinucleotides. His (H) frequently binds trace metals followed by heme. Lys (K) and Arg (R) are most frequently involved in DNA/RNA binding. Trp (W) has very high propensity to bind sugars, while is nearly never observed in interaction with metals. Remaining amino acids do not show pronounced propensities to any specific ligand. From the ligand perspective, metals expectedly demonstrate very low frequencies of interaction with hydrophobic and positively charged residues (Ala, Ile, Leu, Val, Tyr, Phe, and Arg, Lys, respectively).

**TABLE 2:** Amino acid propensities per ligand.

AA	Ligand						
	Coenzyme A	Dinucleotide	DNA/RNA	Heme	Metal	Nucleoside	Sugar
A	−0.01	−0.01	−0.7	−0.3	−1.4	−0.48	−0.99
C	0.37	0.37	−0.32	1.62	2.62	−0.32	−0.32
D	−1.09	−0.17	−0.68	−1.78	1.35	−0.17	0.42
E	−1.21	−1.21	−0.81	−1.91	0.58	−0.52	0.04
F	0.38	−0.31	−0.31	0.79	−1.41	−0.02	−0.02
G	0.31	0.65	−0.2	−0.38	−0.89	0.72	−0.38
H	0.2	0.2	0.49	1.4	2.05	0.2	0.89
I	−0.15	0.03	−0.67	0.03	−1.76	−0.15	−1.07
K	0.18	−0.38	0.88	−0.66	−1.07	0.44	−0.38
L	−0.28	−0.44	−0.84	0.07	−2.23	−0.62	−1.13
M	−0.08	−0.08	−0.08	0.61	−0.77	−0.08	−0.08
N	0.16	0.16	0.34	−0.75	0.16	0.16	0.85
P	−0.14	−0.14	−0.14	−0.14	−1.53	−0.43	−0.43
Q	−0.18	−0.59	0.33	−0.18	−1.28	−0.59	0.1
R	0.54	0.13	1.17	0.28	−1.66	0.28	0.13
S	0.18	0.31	0.18	−0.38	−0.67	0.31	−0.16
T	−0.07	0.4	0.11	−0.07	−0.58	0.4	−0.29
V	−0.01	−0.01	−0.85	−0.16	−1.95	−0.34	−0.85
W	0.37	−0.32	0.37	0.37	NA	−0.32	1.98
Y	0.36	0.13	0.36	0.13	−1.25	−0.16	0.83

The other measure of interest in the context of amino acid covariance analysis is how far apart residues binding the same ligand are located in protein sequence. If ligand binding sites tend to cluster together, i.e., located in close proximity within the primary protein structure, motif scanning methods or machine learning algorithms using relatively short sliding windows could be more efficient in predicting ligand binding sites. On the other hand, if these sites are located far apart, the covariance analysis may appear to be more successful in identifying functional sites, provided that they coevolve. Table 3 presents the distributions of distances between positions of residues binding the same ligand. As can be seen from the table, 23% of metal and sugar binding sites are located 30 or more amino acids apart. Such distances are beyond the sizes of sliding windows commonly employed in prediction methods. The most condensed positions appear for DNA/RNA and heme binding sites, having 76–79% located within five amino acids apart. Nevertheless, all ligands appear to have considerable fractions of binding sites located distantly in primary structure. Therefore, it is pivotal to determine whether such approach as covariance analysis may reveal these remote yet functionally related positions, thereby enhancing the sequence-based functional annotations of proteins.

**TABLE 3:** Percentage distribution of distances between residues binding same ligands.

Distance range (Δaa)	Ligand						
	Coenzyme A	Dinucleotide	DNA/RNA	Heme	Metal	Nucleoside	Sugar
1–4	70.1	68.3	78.7	75.9	51.7	67.8	52.6
5–29	20.3	23.7	15.5	17.9	25.3	17.5	24.5
30–1,000	9.5	8.0	5.8	6.2	23.0	14.8	22.9

### Assessment of CoeViz Metrics

**FIGURE 1:**
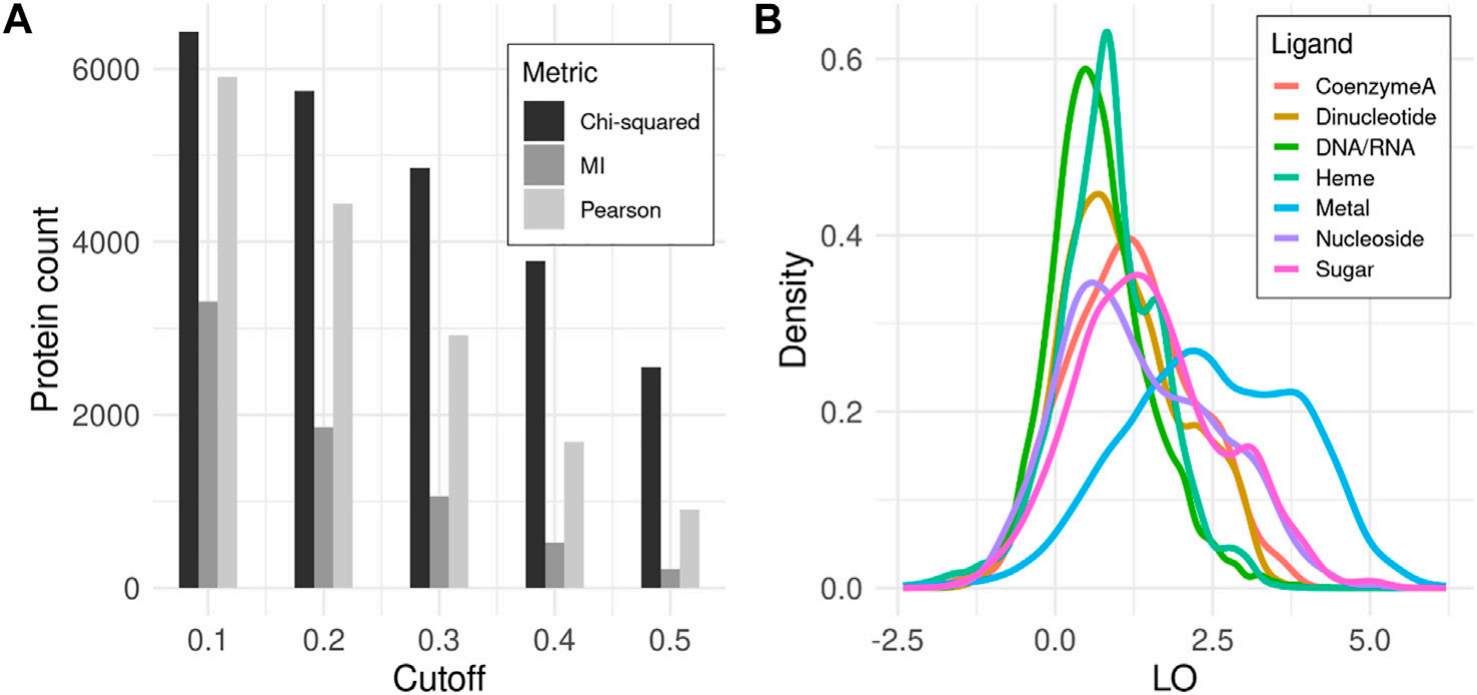
Covariance analysis of functional residues in proteins. **(A)** Counts of proteins that have at least one ligand binding site with coupling to any other position above the given covariance cutoff. **(B)** Density distribution of LO scores for functional residues binding the same ligand among all covarying positions for a given residue (Eq. 9). Here, χ^2^ metric with cutoff ≥ 0.3 is used. LO density distributions for other metrics and cutoffs can be found in Supplementary File S1.

All three covariance metrics implemented in CoeViz have been assessed as to how many proteins contain functional residues that co-vary with any other positions. χ^2^ appears to embrace the highest number of proteins, whereas MI identifies the fewest, encompassing only about 40% proteins in the dataset even at the lowest tested covariance cutoff of 0.1 (Figure 1A). Therefore, subsequent analyses reported here are based on χ^2^ (unless stated otherwise), while the data for other metrics is available in the supplementary materials for comparison (Supplementary File S1). LO scores for covarying residues involved in the same function (Eq. 9) display the highest values within metal binding sites, followed by sugar binding sites. The remaining MFs have lower, yet non-zero LO values (Figure 1B).

To assess whether graph-based cliques differ compared to the nearest neighbors-based covariance grouping, *LO(q)* distributions have also been computed (Eq. 10). The clique-derived LO scores do not show as prominent groupings of the functional sites, beginning to display their enrichment only at cutoffs χ^2^ ≥ 0.3 when the numbers of proteins considered are low (Supplementary File S2). Whether additional residues in cliques represent noise, e.g., from indirect coupling, or actually play supporting roles within corresponding functions is subject to debate, as it is not possible to deduce their relevance in an automated fashion, even from 3D structure. However, such ‘noisy’ members of cliques may potentially be investigated on an individual basis per specific proteins, such as for protein design or mutation analysis. For example, the recent study by Abrusan and Marsh demonstrates that some allosteric sites coevolve with ligand binding sites (Abrusán and Marsh, 2019).

### Comparison With Other In-House Covariance Matrices

To evaluate the effect of graphical LASSO covariance estimation and the size of amino acid alphabet on the enrichment for covarying functional sites, density distributions of LO scores for each ligand have been compared with other possible covariance matrices, including the 2-letter alphabet matrices estimated with graphical LASSO, as well as empirical and estimated with gLASSO 20-letter alphabet-based matrices. LO distribution probabilities *P(LO)* above a given cutoff, along with corresponding counts of proteins and functional residues used to calculate these distributions, are presented in Supplementary File S3.

While it is difficult to directly compare these four types of covariance matrices due to the different number of proteins and residues satisfying any given covariance metric cutoff, there are few major trends clearly visible. The original 2-letter alphabet covariance data (as computed in CoeViz) evidently provides higher enrichment for covarying residues involved in the same MF, also with stronger couplings. The most prominent grouping appears between metal binding sites followed by sugar and nucleoside binding sites. For example, at the χ^2^ ≥ 0.3 cutoff, 3,222 metal binding sites show very high enrichment for other metal binding residues within their covarying environment yielding LO ≥ 3 (37% of 8,707 residues). 2-letter alphabet matrices after gLASSO estimation maintain strong couplings of functional residues, however the number of residues possessing high covariance scores is reduced compared to the CoeViz matrices. On the other hand, 20-letter alphabet matrices appear to yield covariance data for much more functional residues. However, the latter data is very noisy with respect to enrichment for the same ligand binding sites with majority LO scores accumulated around 0.

### Comparison With DCA Methods

To compare with the state-of-the-art methods for covariance analysis, a recently published implementation of DCA has been used–pydca v1 (Zerihun et al., 2020). This Python package computes mean-field (mfDCA) and pseudo-likelihood maximization (plmDCA) inverse statistical approaches of DCA, both in the form of raw matrices and adjusted with APC. We have run pydca to generate matrices of the all four DCA types for all proteins in our benchmark set using the same MSA data generated for our in-house covariance methods, trimming the alignment gaps in the query sequence. As mentioned in Introduction, complex statistical approaches cannot handle large sequences, and we were able to obtain results for the following protein subsets: *n* = 2,578 for mfDCA(raw) and mfDCA(apc); and *n* = 2,495 for plmDCA(raw) and plmDCA(apc). Protein sequence length ranges 30–1,412 aa.

Given these four DCA methods yield covariance scores in different scales, LO probabilities were computed for 9 equally incremented covariance cutoffs between 0 and maximal scores across all assessed proteins (Supplementary File S4). Of the DCA approaches, mfDCA(raw) reveals the most number of couplings between ligand binding sites, though the number of proteins with such covarying sites is low. The APC adjustment improves enrichment for covarying residues binding the same ligand, but it further lowers the number of assessed proteins and residues. For example, at the covariance cutoff ≥ 4, there are 1,595 and 383 covarying functional residues for metal binding sites yielded by mfDCA(raw) and mfDCA(apc), respectively (Supplementary File S4). Overall however, all DCA methods yield strong couplings for the dramatically fewer numbers of functional residues compared to CoeViz (Supplementary File S3). Of note, the direct comparison of LO probability distributions between CoeViz χ^2^ and DCA methods is hindered as there is little overlap of proteins and covarying functional residues for any metric cutoff. Table 4 illustrates this problem for CoeViz and mfDCA(raw) at cutoffs yielding comparable LO distributions.

**TABLE 4:** Intersection of proteins and functional residues for CoeViz χ^2^ and mfDCA(raw) at their 0.3 and 3.0 respective cutoffs yielding comparable LO distributions.

Ligand	CoeViz χ^2^		mfDCA(raw)		Intersection	
	Proteins	Residues	Proteins	Residues	Proteins	Residues
Coenzyme A	99	617	32	260	24	95
Dinucleotide	478	3,817	167	1,176	158	599
DNA/RNA	1,020	11,762	386	5,582	317	2,513
Heme	330	3,866	119	2,118	103	987
Metal	2,336	8,707	831	3,488	496	1,545
Nucleoside	562	3,208	143	657	122	306
Sugar	213	1,364	121	772	60	306

### Example of the CoeViz 2 Analysis

To illustrate how CoeViz 2 can be used to identify groups of functional sites, let us consider a fungal protein Laccase 1 that contains multiple copper binding sites (UniProt ID: Q9Y780). Both from the UniProt annotation and available resolved 3D structure (PDB ID: 1hfu), there are 10 residues binding 3 copper ions. Some of these residues are located hundreds of residues apart in the protein primary structure and therefore hard to identify using a short motif scanning search or mapping from the protein family evolutionary profile. For example, ScanProsite (de Castro et al., 2006) could not pin point any specific residues binding copper, whereas Conserved Domains Database [CDD (Marchler-Bauer et al., 2015)] was able to locate 7 out of 10 metal binding sites.

**FIGURE 2:**
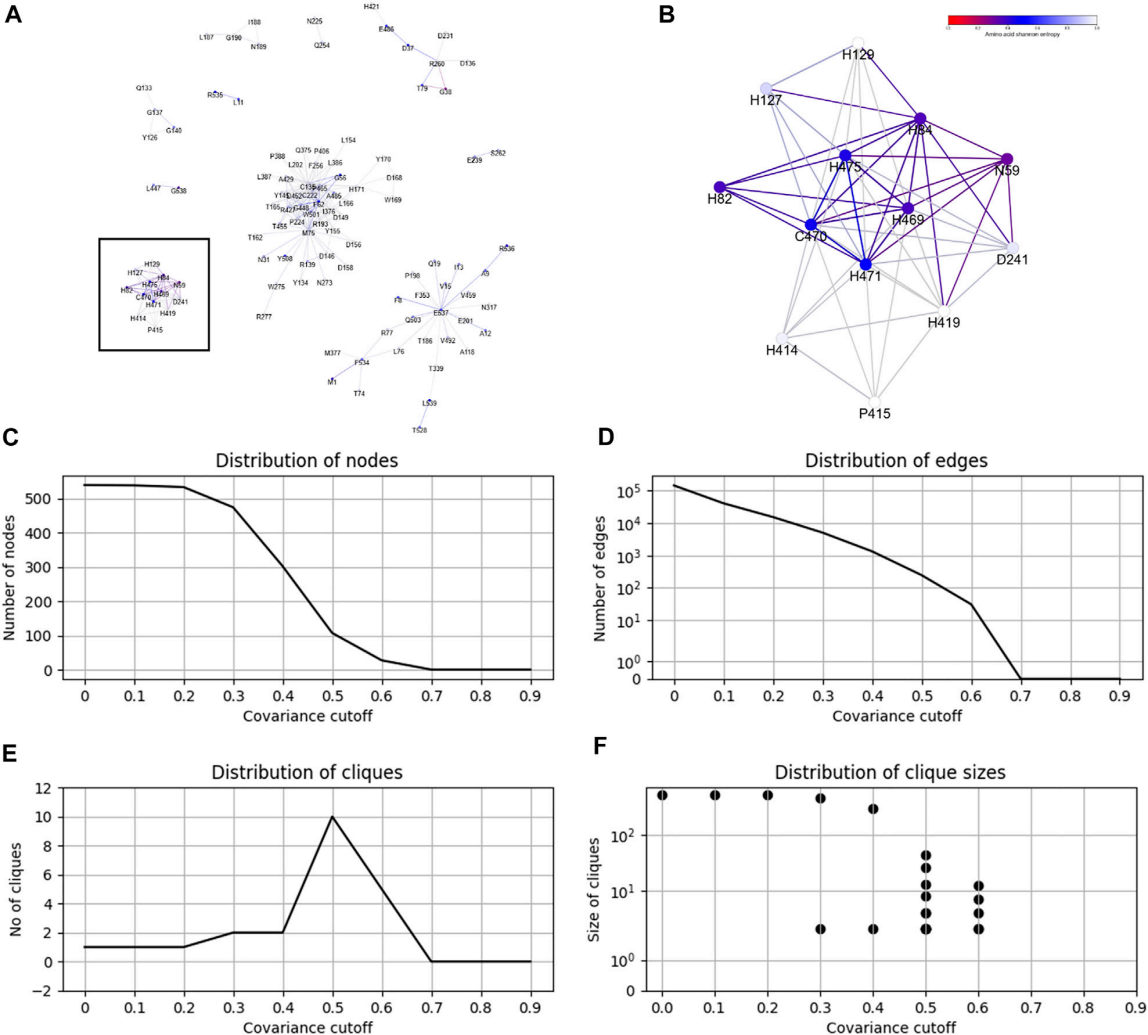
Example of the CoeViz 2 covariance analysis. **(A)** Cliques identified within fungal Laccase 1 (UniProt ID: Q9Y780) using χ^2^ metric based on 2-letter alphabet with cutoff ≥ 0.5 applied. **(B)** A zoom-in view of the clique highlighted by rectangle on panel **A** that contains 9 of 10 residues known to bind copper ions and involved in catalytic activity. Nodes are colored according to their conservation scores expressed by position specific Shannon entropy. **(C–F)** Distributions of graph nodes, edges, cliques and clique sizes per covariance cutoff, respectively. These charts are precomputed for each query to guide the user on the selection of cutoff to visualize a resulting protein graph.

From the CoeViz 2 analysis, 9 of the 10 metal binding sites appear to form a distinct clique from the rest of the graph (Figure 2A, highlighted with rectangle). While the clique contains four other residues (N59, H82, D241, P415), most metal binding sites are interconnected (Figure 2B). Their couplings are also supported by high values of the corresponding LO scores suggesting strong enrichment for residues of the same molecular function (Supplementary File S5). The same protein sequence was also analyzed by the DCA methods (Supplementary File S5). Table 5 compares LO scores for each copper binding site based on covariance data generated by these methods using the highest cutoffs (assessed in previous section) that allow to compute LO for the maximal number of metal binding sites. As can be seen from the table, both plmDCA methods do not yield groupings for 3 to 5 sites even at the lowest assessed cutoff. mfDCA methods miss H84 residue, whereas CoeViz 2 misses H417 at the corresponding chosen cutoffs. On average, the χ^2^ metric yields higher LO scores.

**TABLE 5:** LO scores (Eq. 9) for the copper binding residues in Laccase 1 protein (UniProt ID: Q9Y780) yielded by CoeViz χ^2^ and DCA methods^a^.

Residue	χ^2^ ≥ 0.5	mfDCA(raw) ≥ 2	mfDCA(apc) ≥ 2	plmDCA(raw) ≥ 0.2	plmDCA(apc) ≥ 0.2
H84	3.73	NA	NA	NA	NA
H127	4.09	1.79	2.70	1.38	2.99
H129	4.09	1.61	2.99	1.26	2.99
H414	3.91	2.48	2.99	2.48	2.99
H417	NA	2.59	2.70	2.48	2.99
H419	3.69	2.70	2.70	2.48	2.99
H469	3.69	4.09	4.09	NA	NA
C470	3.69	4.09	4.09	4.09	NA
H471	3.69	4.09	4.09	4.09	NA
H475	3.77	4.09	4.09	NA	NA

a NA–LO score could not be computed due to 0 or undefined argument to logarithm (Eq. 9).

CoeViz 2 provides a web interface for the interactive analysis of residue groupings resulted from the application of different covariance cutoffs. Figures 2C–F illustrates precomputed charts for Laccase 1 to guide the user on the number of nodes, edges, cliques and clique sizes per a given cutoff.

## Discussion

Molecular functions of proteins are multi-pronged and can be viewed at different levels of granularity (refer to molecular function categories used in gene ontology, i.e. GO MF terms). The same protein may exert different MF simultaneously. For example, protein-protein interaction, DNA and small molecule ligand binding are common functions for most transcription factors. Such multi-functionality is generally achieved by the dedicated groups of amino acid residues located at specific positions scattered across protein primary structure but brought in close proximity in tertiary structure. Identification of such groupings of residues involved in the same function remains one of the important problems in computational biology.

The large scale analysis presented in this work clearly suggests that multiple sequence alignment provides a strong coevolutionary signal for positions that are involved in the same MF, such as ligand binding. We would like to point out, though, that the ability of any covariance method in revealing the clusters of coevolving ligand binding sites should be assessed using three parameters at the same time: how strong the covariance scores (couplings) are between the positions of interest, what is the enrichment for residues involved in the same function among the all covarying positions for a given residue at a given covariance cutoff (Eq. 9), and how many proteins and clusters of functional residues can be revealed given the first two parameters. Such complexity of assessment hinders the direct comparison of the presented covariance metrics, especially given that DCA methods yield covariance scores in different scales. Nevertheless, when comparing data in Supplementary Files S1, S3, S4, a few trends are observed. 1) As the covariance cutoff increases, the groupings of functional sites with the same MF become more prominent for any metric, though the number of proteins possessing such strong couplings rapidly drops. 2) While mutual information may be useful for select proteins in this context, other covariance metrics, specifically χ^2^, appear to be more informative and applicable to a dramatically larger number of proteins to reveal such groupings of functional residues. 3) A reduced amino acid alphabet, i.e. 2-letter *vs.* traditionally used 20-letter alphabet, proves to yield less noise in covariance data, especially for metal binding sites. It may be attributed to the fact that only limited types of amino acids predominantly bind metal ions (Table 2). Consequently, contrasting these few types of amino acids against any other types amplifies the signal for coevolving metal binding residues. However, as the example with the Laccase 1 analysis illustrates, the reduced alphabet is not the only reason for high performance of the χ^2^ metric: the protein contains 19 histidines and 5 cysteines, yet the coupling was identified among the metal binding sites only, being confused just with one non-metal binding histidine residue, H82 (Table 5 and Figure 2B). On a large scale, the low confusion rate is further supported by high *P(LO)* values for the large number of residues derived from the CoeViz matrices (Supplementary File S3).

It should be noted that the seven categories of ligands considered here as a proxy to distinguish molecular functions of residues is an approximation, to simplify the problem to be amenable to a large scale analysis. The categories chosen are based on chemical nature of the ligands, their abundance in the benchmark dataset, and available contact annotation in the BioLiP database. The actual molecular functions of the same ligand may vary. For example, copper ions are frequently observed as structural factors facilitating a protein to maintain its 3D fold. But such ions can also be integral part of an active site to catalyze redox reactions. For another example, nuclear receptors have to bind a ligand first in a distinct ligand binding domain, in order to dimerize and subsequently recognize the targeted DNA transcription site by their DNA binding domain. Therefore, residues recognizing a natural ligand may also be considered as those involved in DNA transcription. All these nuances undoubtedly convolute the problem of defining molecular function any given residue is involved in, and skew the assessment of covariance data. However, the goal of this study is to assess whether there is and how strong a coevolutionary signal for functional sites on a large scale. Details on coevolving residues as to why they show couplings can be further explored with such visualization tools as presented here CoeViz 2 in conjunction with other protein annotation resources.

To conclude, we would like to note that CoeViz 2 is not a method for predicting molecular function, but a method for the quick analysis of the protein sequence as a molecular graph and identification of residue couplings with an emphasis on functional residues. Building prediction models based on coevolving residues lies beyond the scope of this work and subject of future studies. Nevertheless, covariance data shows a great promise to be served as input for new methods of amino acid function prediction, augmenting the local environment derived from flanking residues with the information from distant covarying positions.

## Data Availability

The protein sequences (FASTA format) included in the benchmark set can be found at https://doi.org/10.6084/m9.figshare.13578125.v1, and their corresponding ligand binding site annotations projected in to the seven ligand categories can be found at https://doi.org/10.6084/m9.figshare.13578122.v1.

## References

[B1] Abrusán G. Marsh J. A. (2019). Ligand-Binding-Site Structure Shapes Allosteric Signal Transduction and the Evolution of Allostery in Protein Complexes. Mol. Biol. Evol. 36 (8), 1711–1727. 10.1093/molbev/msz093 31004156 PMC6657754

[B2] Baker F. N. Porollo A. (2016). CoeViz: a Web-Based Tool for Coevolution Analysis of Protein Residues. BMC Bioinformatics 17, 119. 10.1186/s12859-016-0975-z 26956673 PMC4782369

[B3] Baker F. N. Porollo A. (2018). CoeViz: A Web-Based Integrative Platform for Interactive Visualization of Large Similarity and Distance Matrices. Data (Basel). 3 (1), 4. 10.3390/data3010004 29423399 PMC5798608

[B4] Berman H. M. Westbrook J. Feng Z. Gilliland G. Bhat T. N. Weissig H. (2000). The Protein Data Bank. Nucleic Acids Res. 28 (1), 235–242. 10.1093/nar/28.1.235 10592235 PMC102472

[B5] de Castro E. Sigrist C. J. A. Gattiker A. Bulliard V. Langendijk-Genevaux P. S. Gasteiger E. (2006). ScanProsite: Detection of PROSITE Signature Matches and ProRule-Associated Functional and Structural Residues in Proteins. Nucleic Acids Res. 34 (Web Server issue), W362–W365. 10.1093/nar/gkl124 16845026 PMC1538847

[B6] de Juan D. Pazos F. Valencia A. (2013). Emerging Methods in Protein Co-evolution. Nat. Rev. Genet. 14 (4), 249–261. 10.1038/nrg3414 23458856

[B7] Dunn S. D. Wahl L. M. Gloor G. B. (2008). Mutual Information without the Influence of Phylogeny or Entropy Dramatically Improves Residue Contact Prediction. Bioinformatics 24 (3), 333–340. 10.1093/bioinformatics/btm604 18057019

[B8] Ekeberg M. Lovkvist C. Lan Y. Weigt M. Aurell E. (2013). Improved Contact Prediction in Proteins: Using Pseudolikelihoods to Infer Potts Models. Phys. Rev. E Stat. Nonlin Soft Matter Phys. 87 (1), 012707. 10.1103/physreve.87.012707 23410359

[B9] Friedman J. Hastie T. Tibshirani R. (2008). Sparse Inverse Covariance Estimation with the Graphical Lasso. Biostatistics. 9 (3), 432–441. 10.1093/biostatistics/kxm045 18079126 PMC3019769

[B11] Jones D. T. Buchan D. W. A. Cozzetto D. Pontil M. (2012). PSICOV: Precise Structural Contact Prediction Using Sparse Inverse Covariance Estimation on Large Multiple Sequence Alignments. Bioinformatics 28 (2), 184–190. 10.1093/bioinformatics/btr638 22101153

[B12] Marchler-Bauer A. Derbyshire M. K. Gonzales N. R. Lu S. Chitsaz F. Geer L. Y. (2015). CDD: NCBI's Conserved Domain Database. Nucleic Acids Res. 43 (Database issue), D222–D226. 10.1093/nar/gku1221 25414356 PMC4383992

[B13] Marks D. S. Hopf T. A. Sander C. (2012). Protein Structure Prediction from Sequence Variation. Nat. Biotechnol. 30 (11), 1072–1080. 10.1038/nbt.2419 23138306 PMC4319528

[B14] Morcos F. Pagnani A. Lunt B. Bertolino A. Marks D. S. Sander C. (2011). Direct-coupling Analysis of Residue Coevolution Captures Native Contacts across many Protein Families. Proc. Natl. Acad. Sci. 108 (49), E1293–E1301. 10.1073/pnas.1111471108 22106262 PMC3241805

[B15] Sustik M. A. Calderhead B. (2012). GLASSOFAST: An Efficient GLASSO Implementation. UTCS Tech. Rep., 1–3.

[B16] Weigt M. White R. A. Szurmant H. Hoch J. A. Hwa T. (2009). Identification of Direct Residue Contacts in Protein-Protein Interaction by Message Passing. Proc. Natl. Acad. Sci. 106 (1), 67–72. 10.1073/pnas.0805923106 19116270 PMC2629192

[B17] Yang J. Roy A. Zhang Y. (2013). BioLiP: a Semi-manually Curated Database for Biologically Relevant Ligand-Protein Interactions. Nucleic Acids Res. 41 (Database issue), D1096–D1103. 10.1093/nar/gks966 23087378 PMC3531193

[B18] Zerihun M. B. Pucci F. Peter E. K. Schug A. (2020). Pydca v1.0: a Comprehensive Software for Direct Coupling Analysis of RNA and Protein Sequences. Bioinformatics 36 (7), 2264–2265. 10.1093/bioinformatics/btz892 31778142

