# Supplementary material for: CoeViz 2: Protein Graphs Derived From Amino Acid Covariance
Source: Front Bioinform. 2021 Jun 24;1:653681. doi: 10.3389/fbinf.2021.653681 (PMC9187035; doi:10.3389/fbinf.2021.653681)
Supplement: Supplementary file 2 [file Table1.DOCX]

Supplementary File S1

LO (Eq. 9 of the main text) density distributions for covarying functional sites based on Chi-squared (χ^2^), Pearson correlation (*r*), and Mutual Information (MI) metrics implemented in CoeViz. Numbers under each plot represent the counts of unique proteins used to compute LO distributions for Coenzyme A, Dinucleotide, DNA/RNA, Heme, Metal, Nucleoside, Sugar binding sites, respectively, at a given cutoff of the corresponding covariance metric.

| Cut­off | *χ*^2^ | | *r* | MI |  |
| --- | --- | --- | --- | --- | --- |
| 0.1 | 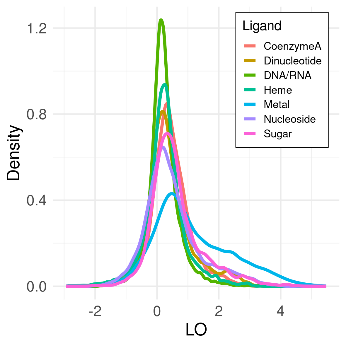  127, 519, 1211, 377, 3476, 708, 332 | | 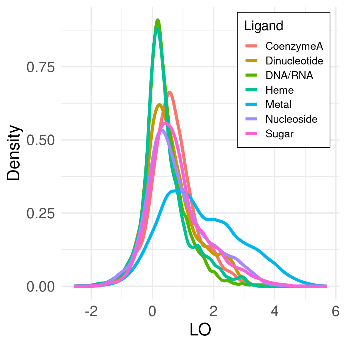  123, 517, 1133, 376, 3091, 655, 294 | 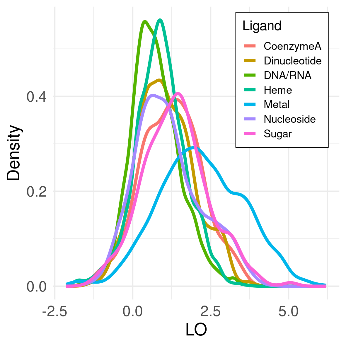  73, 397, 836, 270, 1282, 394, 169 |  |
| 0.2 | 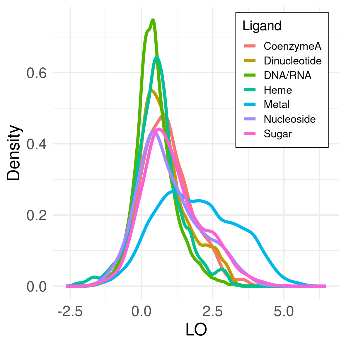  123, 514, 1149, 372, 2904, 659, 275 | | 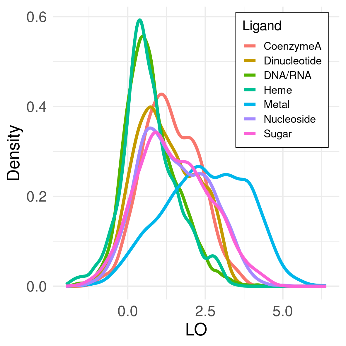  97, 468, 851, 309, 2179, 490, 209 | 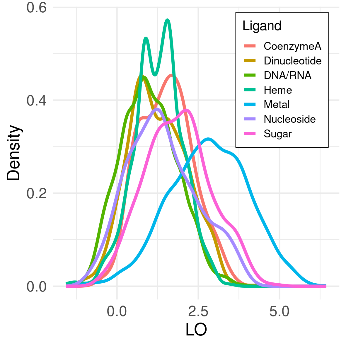  44, 214, 469, 179, 692, 211, 97 |  |
| 0.3 | 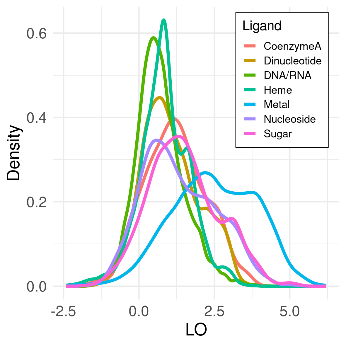  99, 478, 1020, 330, 2336, 562, 213 | | 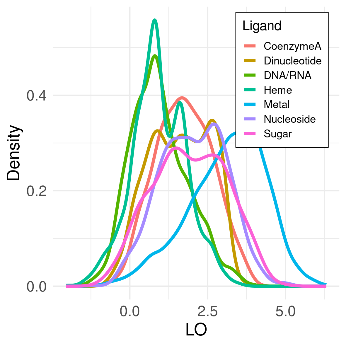  60, 316, 522, 197, 1485, 291, 121 | 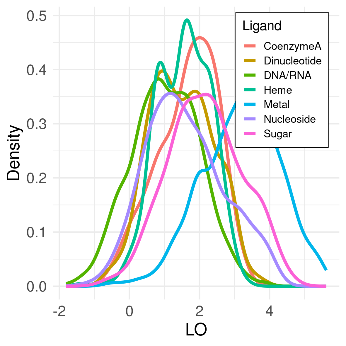  31, 143, 251, 101, 383, 111, 53 |  |
| Cut­off | *χ*^2^ | *r* | | MI | |
| 0.4 | 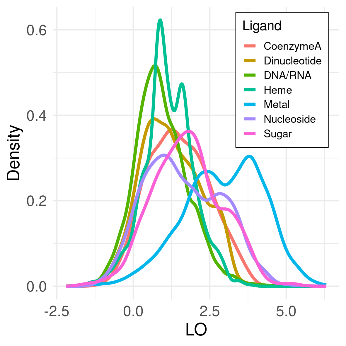  73, 391, 790, 279, 1785, 424, 158 | 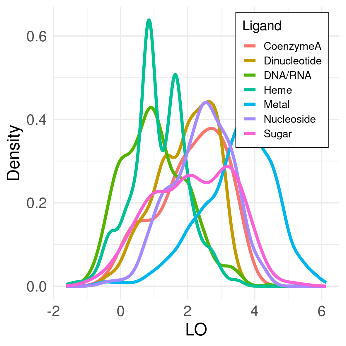  24, 154, 259, 123, 931, 161, 63 | | 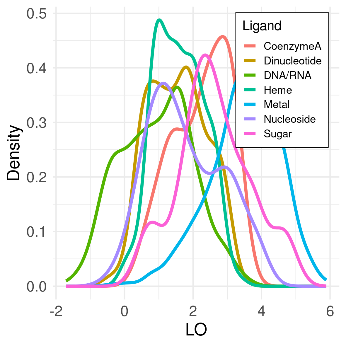  18, 76, 109, 40, 208, 60, 20 | |
| 0.5 | 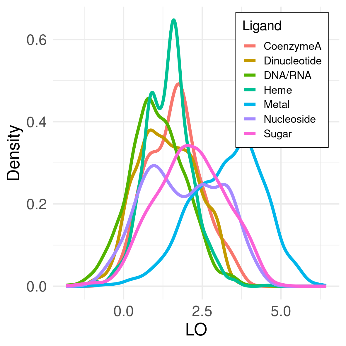  47, 277, 522, 194, 1204, 274, 104 | 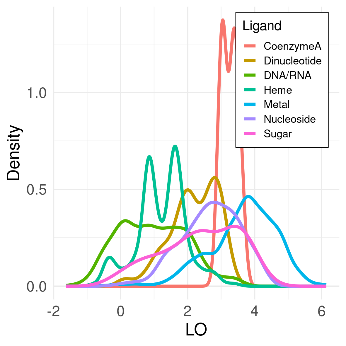  5, 75, 140, 74, 511, 81, 29 | | 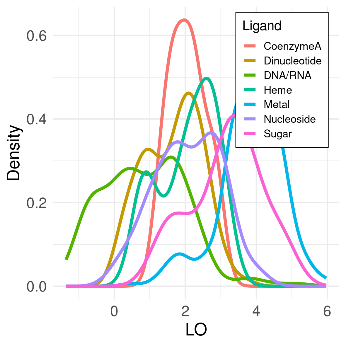  6, 30, 38, 18, 94, 24, 11 | |
| 0.6 | 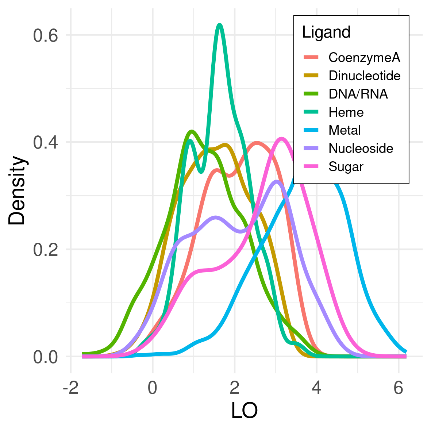  21, 140, 219, 85, 588, 143, 33 | 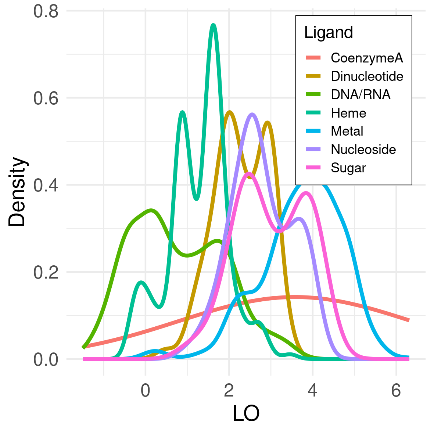  1, 31, 73, 59, 254, 34, 10 | | 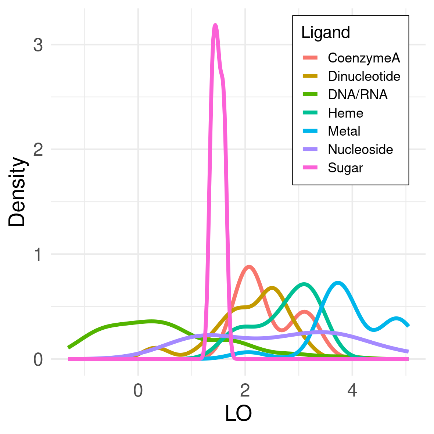  1, 10, 10, 4, 16, 6, 1 | |
