# Supplementary material for: CoeViz 2: Protein Graphs Derived From Amino Acid Covariance
Source: Front Bioinform. 2021 Jun 24;1:653681. doi: 10.3389/fbinf.2021.653681 (PMC9187035; doi:10.3389/fbinf.2021.653681)
Supplement: Supplementary file 3 [file Table2.DOCX]

Supplementary File S2

LO (Eq. 10 of the main text) density distributions for functional sites in cliques based on Chi-squared (χ^2^), Pearson correlation (*r*), and Mutual Information (MI) metrics implemented in CoeViz. Numbers under each plot represent the counts of unique proteins used to compute LO distributions for Coenzyme A, Dinucleotide, DNA/RNA, Heme, Metal, Nucleoside, Sugar binding sites, respectively, at a given cutoff of the corresponding covariance metric.

| Cut­­off | *χ*^2^ | *r* | MI |  |
| --- | --- | --- | --- | --- |
| 0.1 | 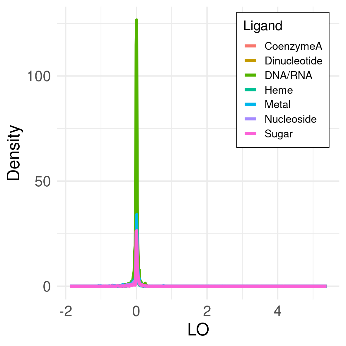  131, 519, 1244, 379, 4345, 720, 390 | 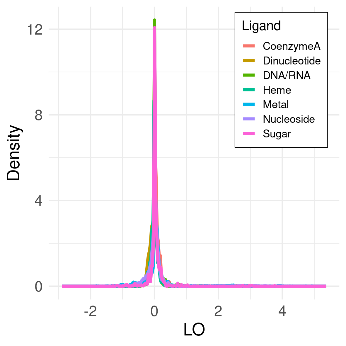  130, 519, 1193, 379, 4026, 703, 371 | 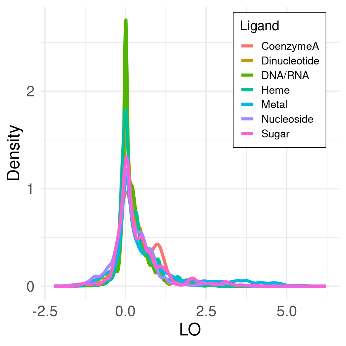  96, 460, 995, 309, 1933, 559, 248 |  |
| 0.2 | 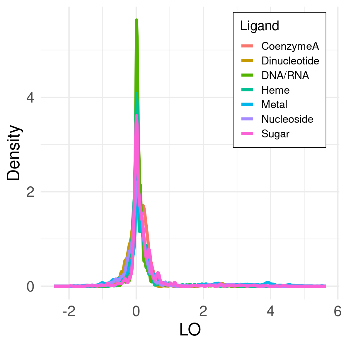  130, 519, 1216, 377, 3742, 712, 354 | 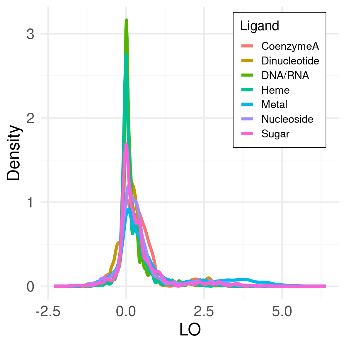  121, 511, 972, 357, 2899, 600, 286 | 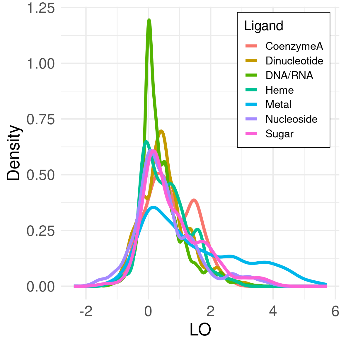  55, 281, 609, 219, 953, 320, 139 |  |
| 0.3 | 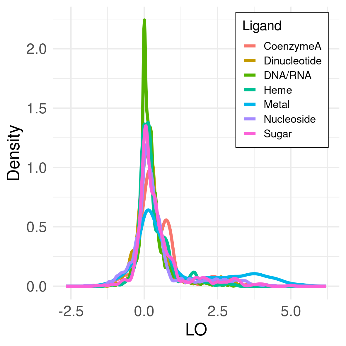  122, 500, 1124, 355, 2969, 666, 297 | 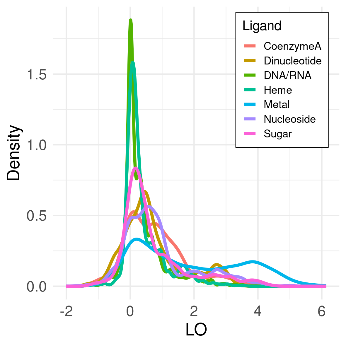  75, 407, 633, 234, 1889, 401, 171 | 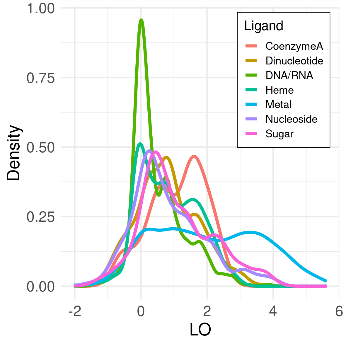  36, 194, 349, 124, 515, 163, 78 |  |
| Cut­­off | *χ*^2^ | *r* | MI | |
| 0.4 | 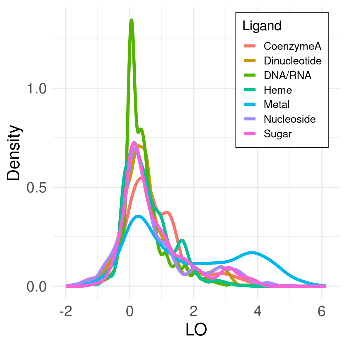  88, 457, 915, 311, 2152, 532, 204 | 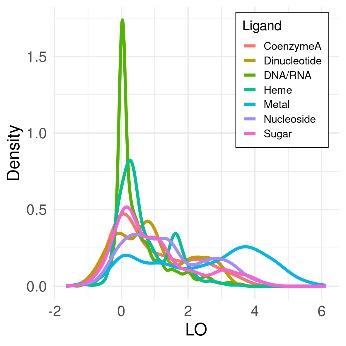  39, 216, 343, 144, 1139, 220, 92 | 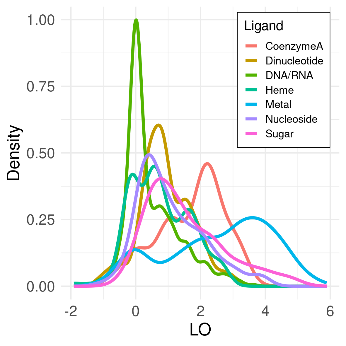  21, 115, 167, 48, 262, 91, 31 | |
| 0.5 | 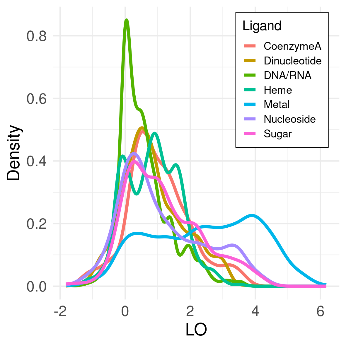  55, 328, 610, 227, 1400, 346, 132 | 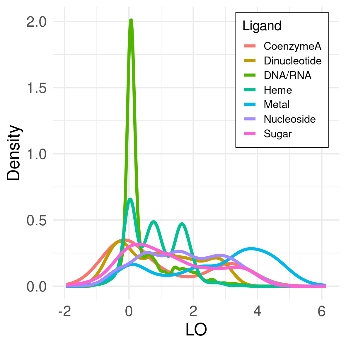  11, 107, 185, 87, 624, 109, 50 | 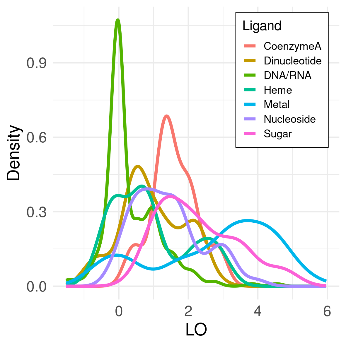  7, 49, 69, 23, 122, 41, 17 | |
| 0.6 | 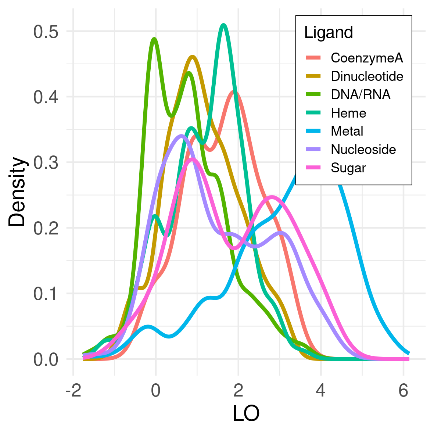  25, 182, 267, 97, 656, 165, 46 | 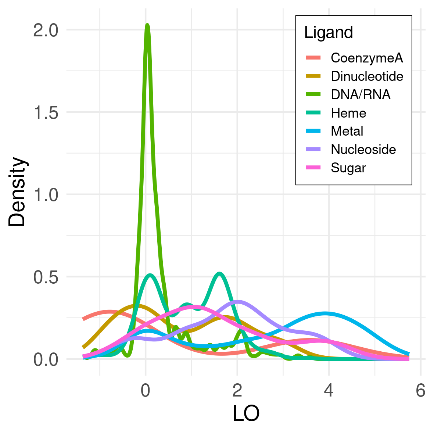  2, 51, 114, 63, 313, 46, 15 | 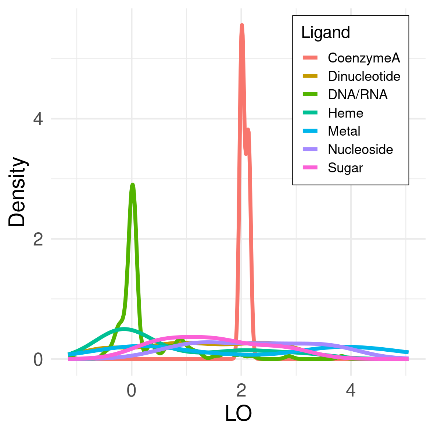  1, 16, 26, 8, 31, 10, 3 | |
